# Deafness from Tobacco Smoking

**Published:** 1883-08

**Authors:** 


### DEAFNESS FROM TOBACCO SMOKING.

Chewing is much less liable to cause these troubles than smoking, because the tobacco smoke comes in contact with a much larger surface than the saliva impregnated with tobacco. Cigarette smoking is the most injurious, because the smoke is so often blown through the nose, and at the same time enters the eustachian tube. The tobacco smoke is laden with fine particles, which gain access to the middle ear and irritate its lining membrane. While this does not admit of actual demonstration, it is rendered highly probable by the fact that disturbances of taste and smell are unquestionably produced in this manner, and are frequently observed in habitual smokers. The long continuance of such an irritation gives rise to a chronic inflammation of the middle ear. The characteristic want of sensibility in the mucous membrane of the throat and nose of smokers who suffer from chronic angina is due to the benumbing influence of tobacco.

